# Monitoring of up to 15 years effects of lipoprotein apheresis on lipids, biomarkers of inflammation, and soluble endoglin in familial hypercholesterolemia patients

**DOI:** 10.1186/s13023-021-01749-w

**Published:** 2021-02-27

**Authors:** J. Víšek, M. Bláha, V. Bláha, M. Lášticová, M. Lánska, C. Andrýs, J. Duintjer Tebbens, Ivone Cristina Igreja e Sá, K. Tripská, M. Vicen, I. Najmanová, P. Nachtigal

**Affiliations:** 1 Metabolism and Gerontology, 3rd Department of Internal Medicine, Faculty of Medicine in Hradec Králové, University Hospital Hradec Králové and Charles University, Hradec Králové, Czech Republic; 2 4th Department of Medicine - Hematology, Faculty of Medicine in Hradec Králové, University Hospital Hradec Králové and Charles University, Hradec Králové, Czech Republic; 3 Department of Immunology and Allergology, Faculty of Medicine in Hradec Králové, University Hospital Hradec Králové and Charles University, Hradec Králové, Czech Republic; 4 Department of Biophysics and Physical Chemistry, Faculty of Pharmacy in Hradec Králové, Charles University, Hradec Králové, Czech Republic; 5 Department of Biological and Medical Sciences, Faculty of Pharmacy in Hradec Králové, Charles University, Akademika Heyrovského 1203, 500 05 Hradec Králové, Czech Republic

**Keywords:** Lipoprotein apheresis, Familial hypercholesterolemia, Lipids, Inflammation, Soluble endoglin

## Abstract

**Background:**

Lipoprotein apheresis (LA) is considered as an add-on therapy for patients with familial hypercholesterolemia (FH). We aimed to analyze the data collected in the last 15 years from FH patients treated with LA, to elucidate the benefit of this procedure with respect to plasma lipids, biomarkers of inflammation, and endothelial dysfunction and soluble endoglin.

**Results:**

14 patients (10 heterozygous FH patients (HeFH), 4 homozygous FH patients (HoFH)) were treated by long-term lipoprotein apheresis. Lipid levels were examined, and ELISA detected biomarkers of inflammation and soluble endoglin. Paired tests were used for intergroup comparisons, and a linear regression model served to estimate the influence of the number of days patients were treated with LA on the studied parameters. LA treatment was associated with a significant decrease of total cholesterol (TC), LDL-C, HDL-C, and apoB, in both HeFH and HoFH patients, after single apheresis and in a long-term period during the monitored interval of 15 years. Biomarkers of inflammation and endothelial dysfunction were reduced for soluble endoglin, hsCRP, and MCP-1, and sP-selectin after each procedure in some HeFH and HoFH patients.

**Conclusions:**

LA treatment up to 15 years, reduced cholesterol levels, levels of biomarkers related to endothelial dysfunction, and inflammation not only after each procedure but also in the long-term evaluation in FH patients. We propose that long-term LA treatment improves lipid profile and endothelial dysfunction in familial hypercholesterolemia patients, suggesting a promising improvement in cardiovascular prognosis in most FH patients.

## Introduction

Familial hypercholesterolemia (FH) is an inherited disorder characterized by elevated plasma levels of low-density lipoprotein (LDL) cholesterol in the absence of hypertriglyceridemia. FH is caused by mutations that reduce the LDL receptor (LDLR) function, with the most common mutations in the LDLR gene itself [1]. Less commonly, the FH phenotype may be caused by mutations in other genes, for example, in apolipoprotein B (apoB), (ligand for the LDLR), PCSK9 (encodes the enzyme proprotein convertase subtilisin/kexin type 9, involved in the regulation of the half-life of the LDL receptor), STAP1 or apoE [2]. Rarely, FH can be caused by autosomal recessive hypercholesterolemia (ARH) with loss of adaptor protein function, encoded by the LDLR adaptor protein 1 gene (LDLRAP1) [3]. Untreated FH is associated with a markedly increased risk of premature cardiovascular disease depending on the specific molecular defect of LDL cholesterol (LDL-C) receptor and coexisting cardiovascular risk factors [4–8].

Lipoprotein apheresis (LA) should be considered as a therapeutic option for patients with severe hypercholesterolemia, who have persistently elevated LDL-C levels, despite pharmacological therapy [9]. It is an extracorporeal elimination technique that removes not only LDL particles but also some other pathogenic lipoproteins such as lipoprotein (a) or triglyceride-rich lipoproteins from the circulation [1]. The main indications for LA are the diagnosis of homozygous FH or heterozygous FH, which are refractory to standard care, intolerance of routine care, and patients with increased lipoprotein(a) resistant to the pharmacotherapy [10]. LA is also a potent therapeutic player with an impact on inflammation and related mediators [11, 12].

Hypercholesterolemia is associated with the induction of inflammation, endothelial dysfunction, and atherosclerosis [13]. There are several well-known circulating biomarkers related to inflammation and endothelial dysfunction, including high-sensitivity C-reactive protein (hsCRP) [14], soluble form of P-selectin (sP-selectin) [15], soluble CD40 ligand (sCD40L) [16], and monocyte chemoattractant protein-1 (MCP-1) [17].

Interestingly, soluble endoglin is a circulating cleaved form of membrane endoglin, a transmembrane glycoprotein related to the function of vascular endothelium [18]. Soluble endoglin might represent an interesting biomarker of increased cholesterol levels since it was shown to be elevated in hypercholesterolemic [19] and FH patients [20]. Also, in an experimental mouse model of atherosclerosis with hypercholesterolemia, soluble endoglin levels were increased before the formation of visible atherosclerosis in the aorta and during the formation of advanced lesions [21–23], suggesting the association of soluble endoglin with hypercholesterolemia and endothelial dysfunction/atherogenesis.

Despite the fact that there are several other long-term studies in patients undergoing regular LA therapy, these studies did not provide a long-term evaluation of selected biomarkers of inflammation, endothelial dysfunction, and soluble endoglin. Thus, in this study, we aimed to evaluate for the first time the effects of long-term LA treatment (up to 15 years) in FH patients on cholesterol levels, biomarkers of inflammation, endothelial dysfunction, and soluble endoglin.

## Results

### Lipoprotein apheresis reduces total cholesterol, LDL-C, HDL-C, and apoprotein B levels.

Treatment with lipoprotein apheresis was associated with a significant decrease of total cholesterol, LDLC, HDLC, apoprotein B after each single lipoprotein apheresis in all patients (both HeFH and HoFH) during the monitored interval of 6–27 years (mean 16.5 ± 4.7 years), median 16.5 years of lipoprotein apheresis therapy (p < 0.001) (Tables 1, 2).

**Table 1:** Baseline characteristics of FH patients

Patient no.	Sex	Type of FH	Mutations	Genotype	Age at the start of LA (y)	LDL-Cat the start of LA (mmol/l)	Duration of LA(y)	CAD	CABG	PCI	AoS	CVD	PAD	DM
1	F	HeFH	−	−	50	10,14	16	+	−	−	−	−	−	−
2	M	HeFH	+	p.Gly324AlafsX46	33	8,95	17	+	−	−	−	+	−	+
3	M	HeFH	+	p.Ser306X	50	6,70	14	+	+	+	−	−	+	+
4	M	HeFH	−	−	60	4,76	20	+	−	−	−	−	−	−
5	M	HeFH	+	p.Cys681X	58	5,47	18	+	_+_	−	−	−	−	−
6	F	HeFH	−	−	52	7,13	14	+	_+_	−	−	−	−	+
7	M	HeFH	+	p.His690ThrfsX19	55	5,60	12	+	−	−	−	+	+	−
8	M	HeFH	+	p.Gly396AlafsX54	39	6,05	13	−	−	−	−	−	−	−
9	F	HeFH	−	−	50	9,50	21	+	+	+	−	+	+	+
10	F	HeFH	−	−	56	4,50	19	−	−	−	−	+	+	−
11	F	HoFH	+	p.Gly592Glu/c.2390-lG>A	14	21,30	17	+	−	+	+	−	−	−
12	F	HoFH	+	p.Asp492Asn/p.Gly592Glu	20	17,50	27	−	−	−	_+_	−	−	−
13	M	HoFH	+	p.Gly592Glu/c.2390-lG>A	22	13,92	16	+	−	−	−	−	−	−
14	F	ARH	+	C.143T.C; p.(Phe48Ser)	58	14,90	7	+	+	+	−	+	−	−

Simultaneously, treatment by LA resulted in a gradual long-term reduction of total cholesterol, LDL-C, and HDL-C in HeFH (significant in 80%, 60%, and 50% of patients, respectively) and HoFH (significant in 100%, 50%, and 50% of patients, respectively) over the studied period (up to 15 years). The average yearly reduction (where the average is computed over those patients exhibiting a statistically significant reduction) was estimated at 0.18 mmol/L for total cholesterol and at 0.18 mmol/L for LDL-C in HeFH. Similarly, in HoFH, the average yearly reduction was estimated to be 0.93 mmol/L and 0.37 mmol/L for total cholesterol and LDL-C, respectively. The yearly average HDL-C level reduction was estimated at 0.04 mmol/L in both HeFH and HoFH, and its clinical significance is discussed later (Table 3).

**Table 2:** The effects of every single lipoprotein apheresis therapy during the monitored interval of 15 years on selected parameters of lipid metabolism, inflammatory biomarkers, and soluble endoglin

Parameter	HeFH (n = 10)			HoFH (n = 4)		
Lipids, mmol/l	Before LA	After LA	Decrease, n (%) patients	Before LA	After LA	Decrease, n (%) patients
TC	5.69 (1.16)	1.93 (0.59)***	10 (100)	7.09 (2.15)	1.95 (0.66)***	4 (100)
LDLC	3.57 (1.19)	0.75 (0.54)***	10 (100)	5.13 (1.87)	0.85 (0.94)***	4 (100)
HDLC	1.34 (0.65)	0.91 (0.33)***	10 (100)	1.51 (0.72)	1.15 (0.39)***	4 (100)
ApoB	1.11 (0.28)	0.28 (0.22)***	10 (100)	1.22 (0.50)	0.18 (0.17)***	4 (100)
Endoglin, ng/mL	6.45 (1.85)	5.56 (1.75)***	8 (80)	6.22 (1.73)	4.85 (1.80)***	3 (75)
CD40L, pg/mL	5323 (2059)	5367 (3093)	4 (40)	5177 (1585)	4239 (1892)	2 (50)
hsCRP, mg/L	2.84 (1.51)	2.39 (1.38)	6 (60)	2.21 (1.36)	1.82 (1.27)	3 (75)
MCP-1, pg/mL	321.8 (99.2)	274.5 (86.1)	6 (60)	265.6 (71.0)	221.4 (68.2)	3 (75)
sP-selectin, ng/mL	148.1 (50.7)	131.7 (53.8)*	7 (70)	145.6 (70.6)	154.3 (212.2)	3 (75)

Data shows the level of parameters before and after each procedure during the time patients underwent LA and the percentage of cases (patients) where change was significant

*p < 0.05, ***p < 0.001

### Lipoprotein apheresis effects on soluble endoglin levels and biomarkers of inflammation and endothelial dysfunction (CD40L, hsCRP, MCP-1, sP-selectin)

Biomarkers of inflammation and endothelial dysfunction have been significantly reduced in some patients treated with LA. In the group of HeFH we found a significant decrease of hsCRP, MCP-1, and sP-selectin after each single apheresis (p < 0.001), and in the group of HoFH, we found a significant decrease of CD40L, MCP-1 (p < 0.001) and hsCRP (p < 0.05) after every single apheresis (p < 0.001) (Table 2). LA resulted in a significant decrease of hsCRP and MCP-1 (both 60% of cases), and sP-selectin (70% of cases) after each procedure in HeFH patients (Table 2). Similarly, in the HoFH group, we found a significant reduction of MCP-1 (75% of cases), CD40L (50% of cases), and hsCRP (75% of cases) (Table 2).

Evaluation of long-term effects of LA showed a significant reduction of sP-selectin in HeFH (40% cases, average yearly reduction 9.92 ng/mL) and a significant decrease of CD40L and hsCRP in HoFH patients (50% and 75% of patients, with average yearly reduction 477 pg/mL and 0.62 mg/L, respectively) (Table 3). Interestingly, even though long-term LA did not significantly affect hsCRP levels in any of the HeFH patients, it reduced hsCRP levels in 75% of cases in HoFH patients (Table 3).

Soluble endoglin levels decreased significantly after each single lipoprotein apheresis (in HeFH from 6.45 + 1.85 ng/mL to 5.56 + 1.75 ng/mL, in HoFH from 6.27 + 1.72 ng/mL to 4.91 + 1.80 ng/mL p < 0.001) (Table 2). This decrease was detected in HeFH patients in 80% of cases and in HoFH in 75% cases (Table 2). In addition, the long-term effects of LA showed a significant reduction of soluble endoglin in HeFH (30% cases, average yearly reduction 0.37 ng/mL) and significant reduction of soluble endoglin in HoFH (25% cases, average yearly reduction 0.52 ng/mL) (Table 3).

A group of 10 heterozygotes of familial hypercholesterolemia (age 50.3 ± 8.4, range 33–60 years) and 4 homozygotes FH (age 28.5 ± 19.9, range 14–58 years) was treated with lipoprotein apheresis by means of immunoadsorption (10 patients) or rheohemapheresis (plasma filtration—4 patients) for a total of 6–27 years (mean 16.5 ± 4.7 years), median 16.5 years. Mann–Whitney Rank Sum Test for intergroup comparisons. The data are shown as mean (SD). Here are the data reported in the number and percentage of patients where a significant effect occurred. *The significance P < 0.01, *** P < 0.001.

HeFH, heterozygous familial hypercholesterolemia; HoFH, homozygous familial hypercholesterolemia; TC, total cholesterol; LDLC, low density lipoprotein cholesterol; HDLC, high density lipoprotein cholesterol; apoB, apoprotein B; CD40L, CD40 ligand; hsCRP, high sensitivity C-reactive protein; MCP-1, monocyte chemoattractant protein-1; sP-selectin, soluble plasma selectin.

A group of 10 heterozygotes of familial hypercholesterolemia (age 50.3 ± 8.4, range 33–60 years) and 4 homozygotes familial hypercholesterolemia (age 28.5 ± 19.9, range 14–58 years) was treated with lipoprotein apheresis by means of immunoadsorption (10 patients) or rheohemapheresis (plasma filtration—4 patients) for a total of 6–27 years (mean 16.5 ± 4.7 years), median 16.5 years. A linear regression model was used to estimate the influence of the number of days when patients were treated with lipoprotein apheresis on the above parameters; here are the data reported in the number and percentage of patients where a significant effect occurred in one column. The next column displays the average (standard deviation) decrease for those patients where a significant effect occurred, expressed in concentration decrease per year.

HeFH, heterozygous Familial hypercholesterolemia; HoFH, homozygous Familial hypercholesterolemia; TC, total cholesterol; LDL-C, low-density lipoprotein cholesterol; HDL-C, high density lipoprotein cholesterol; apoB, apoprotein B; CD40L, CD40 ligand; hsCRP, high sensitivity C-reactive protein; MCP-1, monocyte chemoattractant protein-1; sP-selectin, soluble plasma selectin.

### Multiple linear regression analysis of lipids, biomarkers of inflammation, and endothelial dysfunction (CD40L, hsCRP, MCP-1, sP-selectin) with soluble endoglin

This part of the study aimed to evaluate the possible correlation of soluble endoglin with cholesterol and/or with biomarkers of inflammation and endothelial dysfunction. Despite the significant decrease of lipids (total cholesterol, LDL-C, HDL-C, and apoB) and soluble endoglin in the FH patients treated with LA, we found a significant correlation between soluble endoglin and TC, LDL-C, HDL-C only in 7.1% cases and in apoB only in 14.3% cases (Table 4). Interestingly soluble endoglin correlation was higher with biomarkers of inflammation (MCP-1: 35.7%, hsCRP: 28.6%, CD40L: 14.3%) (Table 5).

**Table 3:** The effects of long-term treatment by LA therapy (average yearly reduction) on selected parameters of lipid metabolism, inflammatory biomarkers, and soluble endoglin

Parameter	HeFH (n = 10)	HeFH (n = 10)	HoFH (n = 4)	HoFH (n = 4)
Lipids, mmol/L	Decrease, n (%)	Mean decrease (SD)/year	Decrease, n (%)	Mean decrease (SD)/year
TC	8 (80)	0.18 (0.08)	4 (100)	0.93 (1.2)
LDL-C	6 (60)	0.18 (0.06)	2 (50)	0.37 (0.19)
HDL-C	5 (50)	0.04 (0.01)	2 (50)	0.04 (0.01)
apoB	1 (10)	0.06 (0)	0 (0)	–
Soluble endoglin, ng/mL	3 (30)	0.37 (0.15)	1 (25)	0.52 (0)
CD40L, pg/mL	3 (30)	714 (193)	2 (50)	477 (122)
hsCRP, mg/L	0 (0)	–	3 (75)	0.62 (0.49)
MCP-1, pg/mL	3 (30)	27.29 (4.84)	1 (25)	15.99 (0)
sP-selectin, ng/mL	4 (40)	9.92 (6.43)	0 (0)	–

Data shows how many patients LA resulted in the change of studied parameters. Moreover, we show an average yearly reduction of levels/biomarkers after LA (where the average is computed over those patients exhibiting a statistically significant reduction)

**Table 4:** Multiple linear regression of parameters of lipid metabolism with soluble endoglin

Lipids, mmol/l	Positive correlation	No correlation	% of positive
TC	1	13	7.1
LDL-C	1	13	7.1
HDL-C	1	13	7.1
apoB	2	12	14.3

Data shows in how many patients we found a significant correlation of soluble endoglin levels with selected lipid parameters

**Table 5:** Multiple linear regression of parameters of inflammation and endothelial dysfunction with soluble endoglin

Biomarker	Positive correlation	No correlation	% of positive
MCP-1	5	9	35.7
hsCRP	4	10	28.6
CD40L	2	12	14.3
sP-selectin	1	13	7.1

Data shows in how many patients we found a significant correlation of soluble endoglin levels with selected biomarkers of inflammation and endothelial dysfunction

A group of ten patients with heterozygous familial hypercholesterolemia (age 50.3 ± 8.4, range 33–60 years) and four with homozygous familial hypercholesterolemia (age 28.5 ± 19.9, range 14–58 years) was treated with LA by means of immunoadsorption (10 patients) or rheohemapheresis (plasma filtration—4 patients) for the period of 16.5 ± 4.7 years (range 6–27 years). Multiple linear regression analysis was used for the correlation.

TC, total cholesterol; LDL-C, low-density lipoprotein cholesterol; HDL-C, high density lipoprotein cholesterol; apoB, apoprotein B.

A group of ten patients with heterozygous familial hypercholesterolemia (age 50.3 ± 8.4, range 33–60 years) and four with homozygous familial hypercholesterolemia (age 28.5 ± 19.9, range 14–58 years) was treated with LA by means of immunoadsorption (10 patients) or rheohemapheresis (plasma filtration—4 patients) for the period of 16.5 ± 4.7 years (range 6–27 years). Multiple linear regression analysis was used for the correlation.

MCP-1, monocyte chemoattractant protein-1; hsCRP, high sensitivity C-reactive protein; CD40L, CD40 ligand; sP-selectin, soluble plasma selectin.

## Discussion

In this paper, we aimed to analyze the data collected in the last 15 years from patients with homozygous or heterozygous FH treated with LA in order to elucidate the benefit of this procedure with respect to plasma lipids, selected biomarkers of inflammation, endothelial dysfunction, and soluble endoglin.

There are two main findings in this study. (i) Treatment with LA was associated with the significant, desirable decrease of total cholesterol and LDL-C not only after every procedure but in the long-term follow-up of up to 15 years, in both HeFH and HoFH patients. (ii) Treatment with LA was associated with the significant reduction of CD40L, MCP-1, and soluble endoglin not only after each procedure but in the long-term evaluation (up to 15 years) in most HeFH and HoFH patients.

The cumulative LDL-lowering effects of combined hypolipidemic therapy predetermined the gradual long-term decrease of total cholesterol and LDL-C in our FH patients. The patients in our study had been treated by LA for a total of 16.5 ± 4.7 years (range 6–27 years). During that long-time follow-up, the guidelines and target values were dramatically changing. Nowadays, the target LDL-C for FH patients with atherosclerotic cardiovascular disease, who are in a very high-risk category, is characterized as ≥ 50% LDL-C reduction from baseline and an LDL-C value < 1.4 mmoL/L, or for FH patients in primary prevention ≥ 50% LDL-C reduction from baseline and an LDL-C value < 1.8 mmoL/L [24]. These target values were quite different when some of our patients started therapy with LA. For example, in the NCEP III guidelines published in the year 2001, the LDL-C target was < 2.6 mmol/L (100 mg/dl) for high-risk, and < 3.4 mmol/L (130 mg/dl) for intermediate-risk patients [25]. Besides, today we have more therapeutic options for FH patients, for example, more potent statins, ezetimibe, PCSK9 inhibitors, or even mipomersen [26]. In addition, lomitapide became available for FH patients and enabled them to lower their LDL-C even more [27]. Some of these novel therapies, namely PCSK9 inhibitors, are much less costly than lipoprotein apheresis (in the Czech Republic less than 10 times). However, because of the lack of insufficient functioning LDL receptors, some of the patients with severe familial hypercholesterolemia, namely homozygous patients, will still not be able to reach LDL cholesterol goals without lipoprotein apheresis.

The main factor for long term reduction of lipid parameters is the change of hypolipidemic therapy over time, as already mentioned above. The hypolipidemic therapy was changing during the time of the study; basically, the dose of statins was increasing, ezetimibe and/or inhibitors of PCSK9 were added based on availability in the Czech Republic and current recommendation for lipid target values. The same is for the management of lipoprotein apheresis. The target values set by the European Atherosclerosis Society and European Cardiology Society were quite different when we started therapy with apheresis and the end of the study period. As we were strictly trying to reach these target lipid values, also the course of more intensive apheresis led to the decreasing lipid values.

Besides that, there is another possible explanation. After the depletion of the rapidly exchangeable pool, represented by, e.g., plasma lipoproteins, erythrocytes, liver, and intestines, recuperation of this pool is mediated by input via an intermediate exchangeable pool (e.g., skin and adipose tissues) and a slowly exchangeable pool (e.g., skeletal muscles and arterial wall). In addition, cholesterol homeostasis will also be corrected by increased cholesterol synthesis and intestinal absorption. However, the therapy, which limits cholesterol synthesis (statins) or decreases cholesterol absorption (ezetimibe), are the most probable reasons why any further transient increase in cholesterol synthesis or absorption was not seen post-apheresis, as discussed previously [28].

LA is a well-established extracorporeal treatment of severe hyperlipoproteinemia. The procedure reduces total and LDL cholesterol very efficiently, which was also confirmed in this study. Several other long-term studies in patients undergoing regular LA therapy evaluate changes in lipids, lipoproteins, clinical events, or compliance ranging from 7 to 22 years [29–32]. However, these studies did not provide a long-term evaluation of selected biomarkers of inflammation, endothelial dysfunction, and soluble endoglin.

Thus, we demonstrate here for the first time that both homozygous and heterozygous patients, treated with apheresis for many years (up to 15 years) have reduced total cholesterol levels not only after each procedure, which is expected, but also gradually each year when they undergo LA.

In this study, LA treatment was associated with a significant decrease in total cholesterol, LDL-C, HDL-C, and apoB after every single apheresis in all patients in the study. Moreover, we showed an average yearly reduction (where the average is computed over those patients exhibiting a statistically significant reduction) in total cholesterol, LDL-C, and HDL-C in both HeFH and HoFH patients. In the patients where both the total cholesterol and LDL cholesterol did not decrease significantly over the long-term period, it did not further increase neither and remained stable. Indeed, we believe that this is an important positive effect of lipoprotein apheresis, which translates into the desired reduction of atherosclerotic cardiovascular complications, as mentioned in the description of the patient group. It is interesting to mention that HDL-C loss during LA occurs primarily because of a reduction in the CE-rich HDL2b subpopulation (approximately 45%). Despite the removal of small amounts of HDL3 (< 20%), selective removal of apoE-containing particles (up to 66%) occurred, and the action of apheresis on apoE-HDL in FH seems to have a primarily atheroprotective character [33].

Besides the reduction of LDL-C and physiology modifications of lipoprotein and lipid metabolism, lipoprotein apheresis may have crucial effects on many other atherogenic factors related to vascular inflammation and endothelial dysfunction [34]. Endothelial dysfunction contributes to the pathogenesis of atherosclerosis, both in the early stages of lesion formation and later during the disease when patients have already developed clinical symptoms [35]. The arterial wall of FH patients is characterized by increased inflammation, which is markedly reduced after LA [36].

Therefore, we aimed to evaluate selected biomarkers of inflammation and endothelial dysfunction, namely CD40L, hsCRP, MCP-1, and sP-selectin. The results showed that hsCRP, MCP-1, sP-selectin are significantly reduced in most of the HeFH patients after apheresis. Similarly, CD40L, hsCRP, MCP-1 were decreased in HoFH patients. These data clearly show the benefit of the apheresis not only on the levels of plasma lipids but also on the levels of inflammation biomarkers. These results are in line with several papers showing that a single procedure results in the reduction of hsCRP, MCP-1, sP-selectin [37, 38]. We have previously shown that the levels of sP-selectin and MCP-1 decreased significantly after apheresis and could be used as another marker showing the effectivity of the extracorporeal LDL-C elimination (immediately after the procedure), and may serve as a marker of the therapy efficacy [17]. Sampietro et al*.* demonstrated the reduction of adhesion molecules by dextran sulfate columns (sICAM and sELAM—soluble intercellular and soluble leukocyte cell adhesive molecules) [39]. Empen et al*.* 2002 found a decrease in E-selectin levels, VCAM-1, and ICAM-1 by direct absorption of lipids, dextran sulfate adsorption, or heparin extracorporeal low-density lipoprotein precipitation [40]. Pulawski et al*.* 2002 found reduced plasma levels of sVCAM-1, sICAM-1, and P-selectin during heparin extracorporeal low-density lipoprotein precipitation (HELP) [41]. Our results do not mean that decreased levels of inflammatory biomarkers were associated with decreased frequency of infectious complications (e.g., infections). We proposed that long-term LA treatment improves lipid profile, microinflammation and endothelial dysfunction associated with atherosclerosis in familial hypercholesterolemia patients, suggesting a promising improvement in cardiovascular prognosis in most FH patients. Thus, we suggest that familial hypercholesterolemia patients will benefit from long-term apheresis procedure by the reduction of microinflammation, which might be important for the prevention of atherosclerotic cardiovascular events.

We extended the observation of these biomarkers of inflammation and endothelial dysfunction for the 15 years follow-up in this study. In addition, we measured the levels of selected biomarkers before each apheresis and evaluated whether the levels of these biomarkers are reduced after several years of apheresis when compared to the beginning of the procedure. We showed that sP-selectin and MCP-1 were significantly reduced in some HeFH patients, and hsCRP was decreased in most of the HoFH patients. Indeed, the patients aged during the long-term period of observation throughout our study (16.5 ± 4.7 years (range 6–27 years)); therefore, we cannot exclude that some biomarkers of endothelial dysfunction and inflammation may change with age. On the other hand, we propose that it is unlikely that biomarkers of endothelial dysfunction and inflammation would be decreased with age. Thus, we suggest that familial hypercholesterolemia patients will benefit from long-term apheresis procedure by the reduction of inflammation, which might be important for the prevention of cardiovascular events. We suggest that the genotype influences may be one of the probable explanations for the differences in the reduction of inflammatory parameters between HeFH and HoFH. We think that genotype influences may be one of the probable explanations for the differences in the reduction of inflammatory parameters between HeFH and HoFH. In addition, as recently published, lipoprotein apheresis has an impact on plasma-circulating levels of miRNAs associated with lipid homeostasis and vascular status. There may be a potential relationship between mutations within the LDLR and several miRNA, but these results should be verified in a larger study cohort [42]. Soluble endoglin might represent a novel and exciting biomarker in cardiovascular disorders. It is released into the circulation during hypercholesterolemia endothelial dysfunction [43], atherosclerosis [23], type 2 diabetes mellitus, and arterial hypertension [44]. It was also demonstrated that high levels of soluble endoglin contribute to the development of arterial hypertension [45], and when combined with hypercholesterolemia, it can aggravate endothelial dysfunction [46]. This implies that the reduction of soluble endoglin might be beneficial with respect to the function of vascular endothelium and the development of cardiovascular complications in FH patients. It was demonstrated that soluble endoglin levels are higher in patients with familial hypercholesterolemia when compared to healthy controls [20]. Moreover, soluble endoglin levels were reduced after a series of two aphereses, suggesting that soluble endoglin serves as an indicator of a beneficial and sufficient procedure in patients with familial hypercholesterolemia [20].

In this study, we extended this information by the fact that soluble endoglin is reduced after each apheresis in most of HoFH and HeFH patients. Moreover, soluble endoglin levels were reduced in more HeFH patients compared to other traditional biomarkers, namely CD40L, hsCRP, MCP-1, and sP-selectin. Interestingly, soluble endoglin levels were also reduced in some HeFH patients when the effect of long-term apheresis was evaluated. Further analysis revealed soluble endoglin correlation with biomarkers of inflammation (rather than cholesterol levels), including MCP-1 and hsCRP in some patients, suggesting soluble endoglin reflects endothelial inflammatory status in some patients treated with apheresis.

It is of interest to mention that the measurement of samples before and after passage through the elimination media did not demonstrate any non-specific capture of soluble endoglin and other biomarkers in the adsorption and filtration columns [20].

### Study limitations

Our study had a small sample size, especially in the treatment group, and we did not incorporate a control treatment arm in this study. The small number of participants in the present study may affect the accuracy of our results. The sample size was too small, and thus our study cannot appropriately identify whether LA indeed decreased the levels of different biomarkers of inflammation. These findings should be verified by multicenter prospective studies. In addition, we cannot exclude the influence of concomitant treatment besides lipoprotein apheresis (statins, ezetimibe, PCSK9 inhibitors) on the reduction of lipid and/or inflammatory parameters. Furthermore, since the lipoprotein apheresis treatment technique is carried out only in a few big medical centers, many FH patients are unable to receive apheresis treatment, resulting in a particular bias in patient selection. Our study's strength is the collection of a large set of data comprising the long-term treatment with LA for a total of 15 years.

## Conclusions

We demonstrate here for the first time that LA treatment for up to 15 years reduces total cholesterol levels in both homozygous and heterozygous patients diagnosed with familial hypercholesterolemia, not only after every procedure but also in the long-term perspective as well. Moreover, LA results in a decrease of biomarkers related to endothelial dysfunction and inflammation (hsCRP, MCP-1, and soluble endoglin) not only after each procedure but also in the long-term evaluation (at least in most patients). We propose that long-term LA treatment improves lipid profile and endothelial dysfunction in familial hypercholesterolemia patients, suggesting potential benefits against the development of cardiovascular complications.

## Methods

### Subjects

The protocol was carried out according to the Declaration of Helsinki. All examined individuals were Caucasians, and all signed informed consent forms, which, together with the protocol of the study, were approved by the institute's ethics committee.

The demographic, anthropometric, and clinical characteristics of patients at baseline are summarized in Table 1. FH patients treated by long-term LA were included in our study. There were 14 patients (eight male and six female), ten diagnosed with heterozygous FH (HeFH, age 50.3 ± 8.4, range 33–60 years), and four with homozygous FH (HoFH, age 28.5 ± 19.9, range 14–58 years). LDL-C before the start of LA was 6.88.3 ± 2.01 (range 4.50—10.14) mmol/l in HeFH patients and 16.91 ± 3.29 (range 13.92–21.30) mmol/l in HoFH patients. The characteristics and clinical phenotype of FH under combined long-term LA/hypolipidemic therapy were described previously [47]. DNA-based evidence of a mutation in the *LDLR* gene was the criterion for the diagnosis of HoFH: three (75%) homozygous FH patients were classified as double heterozygous, and one (25%) patient with ARH was homozygous for the *LDL receptor adaptor protein 1* gene. For HeFH diagnosis: Five (50%) HeFH patients had DNA-based evidence of a mutation in the *LDLR* gene. No mutation was confirmed so far in the other five (50%) HeFH patients; these patients were selected according to Dutch Lipid Network Criteria [5]. None (0%) of the patients had proven a mutation in the *APO-B* or *PCSK9* gene. All patients had confirmed atherosclerotic lesions determined by the ultrasonographically measured carotid artery intima-media thickness and/or by coronarography. Patients with diseases and conditions known to increase the concentration of inflammatory markers, such as acute infection, chronic inflammatory, and autoimmune diseases, as well as malignancies, were excluded. All patients were treated daily with high-dose statins (rosuvastatin 40 mg or atorvastatin 80 mg) at the last assessment. The hypolipidemic therapy was changing during the time of the study; basically, the dose of statins was increasing, ezetimibe and/or inhibitors of PCSK9 were added based on availability in the Czech Republic and current recommendation for lipid target values. Thus, one HeFH patient (7%) was treated in combination with fenofibrate (160 mg), two patients (14%) in combination with bile acid-binding resins (6 mg), all patients (100%) in combination with ezetimibe (10 mg), and seven patients (50%) with PCSK9 inhibitors (alirocumab 150 mg and/or evolocumab 140 mg every two weeks) at the end of the sampling period (4 HoFH, 3 HeFH patients) (range of treatment 5 months—6 years).

F, female; M, male; FH, familial hypercholesterolemia; HeFH, heterozygous familial hypercholesterolemia; HoFH, homozygous familial hypercholesterolemia; ARH, autosomal recessive hypercholesterolemia; LA, lipoprotein apheresis; LDL-C, low-density lipoprotein cholesterol; CAD, coronary heart disease; CABG, coronary artery bypass graft surgery; PCI, percutaneous coronary intervention; AoS, aortic valve stenosis; CVD, cerebrovascular disease; PAD, peripheral artery disease; DM, diabetes mellitus.

Twelve (86%) patients have been without progression of cardiovascular disease (CVD), one HoFH patient (7%) underwent aortocoronary bypass surgery for coronary heart disease at the age of 27 years, one HoFH patient (7%) underwent aortic valve replacement for aortic stenosis, one HoFH patient (7%) survived a recurrent ischemic stroke at the age of 64 years. However, the last patient was treated by lipoprotein apheresis for the shortest interval (7 years), started late (at the age of 58 years), and was treated only once a month because of the apheresis center’s distance [48]. No patient died during the study period; however, six months after completing the study, one HoFH patient died due to the artificial aortic valve thrombosis, associated with acute myocardial infarction during pregnancy, at the age of 32.

### Lipoprotein apheresis

Patients were treated with LA immunoadsorption (10 patients) or rheohemapheresis (plasma filtration—4 patients) for the period of 16.5 ± 4.7 years (range 6–27 years). However, in this study, we only include data from the last 15 years (2004–2019). The rate of side effects of long-lasting lipoprotein apheresis in our patients with familial hyperlipoproteinemia did not exceed 6.26% and was subsequently resolved by standard symptomatic therapy [49]. Plasma separation was carried out using a Cobe-Spectra or Optia continuous centrifugal separator (Terumo, Likewood, Co, USA) in 9 patients. An adsorption–desorption automatic device (Adasorb, Medicap, Germany) controlled repeated fillings and washings of Lipopak adsorbers used for the reduction of LDL cholesterol (Pocard, Moscow, Russia). In 2 patients, Lipocollect adsorbers (Medicollect, Germany) were used. 3 patients simultaneously received long-term rheohemapheresis therapy due to hypercholesterolemia and increased levels of fibrinogen. Plasma was collected using a Cobe-Spectra or Optia separator following high-speed centrifugation. Afterward, it went through a "second stage" consisting of a filter (Evaflux 4A, Kawasumi, Tokyo, Japan) with hollow fibers of ethylene–vinyl alcohol with 0.03 mm pores. We used the CF-100 (Infomed, Geneva, Switzerland) as a secondary device. The plasma flow was continuous, and anticoagulation was secured with heparin. The basic volume of processed plasma was 1.5 times the body volume, as calculated using the Cobe separator. Initially, the LDL-C target level was considered to be less than 1 mmol/L after lipoprotein apheresis. We now consider a target level of 0.5 mmol/L to be more beneficial to the patient.

### Plasma samples and blood analysis

The patients were sampled throughout their treatment by LA every six months. Blood samples were collected immediately before and after LA or hemorheopheresis in EDTA-containing tubes and centrifuged within 30 min at 1500G for 15 min at room temperature. Plasma samples were aliquoted and stored at − 80 °C before the proteomic analysis. The analysis was performed periodically with a consistent methodology. Data from the last 15 years (2004–2019) were used. Even though older samples were available, the data were not included in this analysis because of the incomparable methodology.

TC, LDL-C, HDL-C, apoprotein B, and triglycerides were determined using a commercial kit with a Modular Roche analyzer, as previously described [50].

Analyses investigating whether biomarkers of inflammation and soluble endoglin are non-specifically adsorbed in columns or filters were performed previously [17, 20]. Briefly, two columns used for each of eight patients (total of 16 columns) and four Evaflux 4A filters (for capture at hemorheopheresis) were examined. Levels of investigated parameters were measured in plasma flowing in and out of the columns, in the first half of the first adsorption cycle, in each column. In the filtration method, levels of investigated parameters were determined in front of and behind the filter.

### Biomarkers of inflammation and soluble endoglin

The levels of hsCRP were assessed by immunonephelometry with analyzer IMMAGE 800 (Beckman, USA), and results were expressed in milligrams per liter (mg/L) of serum with a detection limit of 1.0 mg/L.

The serum concentrations of the human soluble form of P-selectin were determined by sandwich enzyme-linked immunosorbent assay technique (ELISA) with Human Quantikine P-Selectin/CD62P ELISA commercial kit (R&D Systems, MN, USA) according to the manufacturer’s instructions. The limit of detection of sP-Selectin was 0.05 ng/mL. Samples were diluted at 1:20. Absorbance values were measured at 450 nm/620 nm by Multiskan RC ELISA reader (Thermo Fisher Scientific, MA, USA).

Concentrations of soluble endoglin (CD105) were assessed in serum samples with ELISA using the Quantikine Human Endoglin/CD105 ELISA kit (R&D Systems, MN, USA) according to the manufacturer’s instructions. Samples were undiluted. The sensitivity of the kit was 0.007 ng/mL. The absorbance values were measured at 450 nm with a Multiskan RC ELISA reader (Thermo Fisher Scientific, MA, USA).

The level of MCP-1 was evaluated by ELISA using Quantikine Human CCL2/MCP-1 ELISA kit (R&D Systems, MN, USA) according to the manufacturer’s instructions. The concentration was expressed in picograms per milliliter (pg/mL) of serum, with a detection limit of 1.7 pg/mL. Samples were diluted twice (1:1) with specific diluent. The absorbance values were measured at 450 nm by a Multiskan RC ELISA reader (Thermo Fisher Scientific, MA, USA).

The levels of CD40 ligand (CD40L) were detected by ELISA kit Quantikine Human CD40 Ligand/TNFSF5. The kit was manufactured by R&D Systems, MN, USA. The assay was run according to the instructions for use provided by the manufacturer. Absorbance was measured at 450 nm with the microplate reader Multiskan RC ELISA reader (Thermo Fisher Scientific, MA, USA). Serum samples were 1:5 diluted, and the concentration of CD40L was expressed in pg/mL of serum, with a detection limit of 4.2 pg/mL.

### Other laboratory parameters

Besides the above-mentioned laboratory analyses, we have performed other multiple investigations (lipoprotein (a), primary hemostasis, the involvement of thrombocytes etc.), which were published previously and are therefore not reported in this manuscript [51–53].

### Statistical analyses

Apart from absolute and relative patient frequencies, data are presented as mean (standard deviation). Wilcoxon matched-paired signed-rank tests were used for intergroup comparisons. Linear regression was performed to estimate the influence of the number of days patients are treated with LA on the parameters mentioned above. Multiple linear regression was used to assess whether soluble endoglin is influenced by the factors of cholesterol and lipoprotein levels and/or biomarkers of inflammation and endothelial dysfunction. A value of P < 0.05 was the minimum requirement for statistical significance. GraphPad Prism 8.0 software (La Jolla, CA, USA), JMP (2012 SAS Institute, Inc.), and SigmaPlot (2012 Systat Software, Inc.) statistical software were used for the statistical analyses.

## Data Availability

All data generated or analyzed during this study are included in this published article (and its supplementary information files).

## References

[1] Kastelein JJP, Reeskamp LF, Hovingh GK (2020). Familial hypercholesterolemia: the most common monogenic disorder in humans. J Am Coll Cardiol.

[2] Abifadel M, Elbitar S, El Khoury P (2014). Living the PCSK9 adventure: from the identification of a new gene in familial hypercholesterolemia towards a potential new class of anticholesterol drugs. Curr Atheroscler Rep.

[3] Alnouri F, Athar M, Al-Allaf FA (2018). Novel combined variants of LDLR and LDLRAP1 genes causing severe familial hypercholesterolemia. Atherosclerosis.

[4] Kim YR, Han KH (2013). Familial hypercholesterolemia and the atherosclerotic disease. Korean Circ J.

[5] Nordestgaard BG, Chapman MJ, Humphries SE (2013). Familial hypercholesterolaemia is underdiagnosed and undertreated in the general population: guidance for clinicians to prevent coronary heart disease: consensus statement of the European Atherosclerosis Society. Eur Heart J.

[6] Cenarro A, Etxebarria A, de Castro-Orós I (2016). The p. Leu167del mutation in APOE gene causes autosomal dominant hypercholesterolemia by down-regulation of LDL receptor expression in hepatocytes. J Clin Endocrinol Metab.

[7] Civeira F, Plana N (2017). Treatment of heterozygous familial hypercholesterolemia in children and adolescents: an unsolved problem. Revista Española de Cardiología (Engl Ed).

[8] Creider J, Hegele R. Clinical evaluation for genetic and secondary causes of dyslipidemia. In: Elsevier (ed) Clinical lipidology, 2nd ed. Philadelphia (USA), 2015, p. 128–34.

[9] Bambauer R, Bambauer C, Lehmann B, et al. LDL-apheresis: technical and clinical aspects. Sci World J; 2012. 10.1100/2012/314283 PMC3361163 22654591

[10] Blaha V, Blaha M, Lanska M (2017). Lipoprotein apheresis in the treatment of dyslipidemia–the Czech Republic Experience. Physiol Res.

[11] Stefanutti C, Zenti MG (2018). Lipoprotein apheresis and PCSK9-inhibitors. Impact on atherogenic lipoproteins and anti-inflammatory mediators in familial hypercholesterolaemia. Curr Pharm Des.

[12] Blaha V, Blaha M, Solichova D (2017). Antioxidant defense system in familial hypercholesterolemia and the effects of lipoprotein apheresis. Atheroscler Suppl.

[13] Chepelenko GV (2003). Pathogenesis of atherosclerosis in patients with lipid metabolism disturbances: hypothesis on cholesterol utilization and atheromatous plaque formation. Angiol Sosud Khir.

[14] Ridker PM, Silvertown JD (2008). Inflammation, C-reactive protein, and atherothrombosis. J Periodontol.

[15] Hajilooi M, Sanati A, Ahmadieh A (2004). Circulating ICAM-1, VCAM-1, E-selectin, P-selectin, and TNFRII in patients with coronary artery disease. Immunol Invest.

[16] Szabolcs MJ, Cannon PJ, Thienel U (2000). Analysis of CD154 and CD40 expression in native coronary atherosclerosis and transplant associated coronary artery disease. Virchows Arch.

[17] Blaha M, Krejsek J, Blaha V (2004). Selectins and monocyte chemotactic peptide as the markers of atherosclerosis activity. Physiol Res.

[18] Schoonderwoerd MJA, Goumans MTH, Hawinkels L (2020). Endoglin: beyond the endothelium. Biomolecules.

[19] Blann AD, Wang JM, Wilson PB (1996). Serum levels of the TGF-beta receptor are increased in atherosclerosis. Atherosclerosis.

[20] Blaha M, Cermanova M, Blaha V (2008). Elevated serum soluble endoglin (sCD105) decreased during extracorporeal elimination therapy for familial hypercholesterolemia. Atherosclerosis.

[21] Vicen M, Vitverova B, Havelek R (2019). Regulation and role of endoglin in cholesterol-induced endothelial and vascular dysfunction in vivo and in vitro. FASEB J.

[22] Strasky Z, Vecerova L, Rathouska J (2011). Cholesterol effects on endoglin and its downstream pathways in ApoE/LDLR double knockout mice. Circ J.

[23] Rathouska J, Vecerova L, Strasky Z (2011). Endoglin as a possible marker of atorvastatin treatment benefit in atherosclerosis. Pharmacol Res.

[24] Mach F, Baigent C, Catapano AL (2019). 2019 ESC/EAS Guidelines for the management of dyslipidaemias: lipid modification to reduce cardiovascular risk. Atherosclerosis.

[25] Adults EPoDEaToHBCi. Executive summary of the third report of the national cholesterol education program (NCEP) expert panel on detection, evaluation, and treatment of high blood cholesterol in adults (adult treatment panel III). JAMA: J Am Med Assoc. 2001;285: 2486–97. 10.1001/jama.285.19.2486. 10.1001/jama.285.19.2486 11368702

[26] Parham JS, Goldberg AC (2019). Mipomersen and its use in familial hypercholesterolemia. Expert Opin Pharmacother.

[27] Bruckert E, Gallo A (2017). Is lomitapide a life-saving drug in homozygous familial hypercholesterolemia. Eur J Prev Cardiol.

[28] Kroon AA, Van’Hof MA, Demacker PN (2000). The rebound of lipoproteins after LDL-apheresis Kinetics and estimation of mean lipoprotein levels. Atherosclerosis.

[29] Beliard S, Gallo A, Duchene E (2018). Lipoprotein-apheresis in familial hypercholesterolemia: Long-term patient compliance in a French cohort. Atherosclerosis.

[30] Kolovou G, Hatzigeorgiou G, Mihas C (2012). Changes in lipids and lipoproteins after selective LDL apheresis (7-year experience). Cholesterol.

[31] Makino H, Harada-Shiba M (2003). Long-term effect of low-density lipoprotein apheresis in patients with homozygous familial hypercholesterolemia. Therapeutic Apher Dial.

[32] Masaki N, Tatami R, Kumamoto T (2005). Ten-year follow-up of familial hypercholesterolemia patients after intensive cholesterol-lowering therapy. Int Heart J.

[33] Orsoni A, Saheb S, Levels JH (2011). LDL-apheresis depletes apoE-HDL and pre-beta1-HDL in familial hypercholesterolemia: relevance to atheroprotection. J Lipid Res.

[34] Neumann CL, Schulz EG, Hagenah GC (2013). Lipoprotein apheresis – More than just cholesterol reduction?. Atheroscler Suppl.

[35] Higashi Y, Noma K, Yoshizumi M (2009). Endothelial function and oxidative stress in cardiovascular diseases. Circ J.

[36] van Wijk DF, Sjouke B, Figueroa A (2014). Nonpharmacological lipoprotein apheresis reduces arterial inflammation in familial hypercholesterolemia. J Am Coll Cardiol.

[37] Utsumi K, Kawabe M, Hirama A (2007). Effects of selective LDL apheresis on plasma concentrations of ICAM-1, VCAM-1 and P-selectin in diabetic patients with arteriosclerosis obliterans and receiving maintenance hemodialysis. Clin Chim Acta.

[38] Kobayashi S, Oka M, Moriya H (2006). LDL-apheresis reduces P-Selectin, CRP and fibrinogen – possible important implications for improving atherosclerosis. Ther Apher Dial.

[39] Sampietro T, Tuoni M, Ferdeghini M (1997). Plasma cholesterol regulates soluble cell adhesion molecule expression in familial hypercholesterolemia. Circulation.

[40] Empen K, Otto C, Brödl UC (2002). The effects of three different LDL-apheresis methods on the plasma concentrations of E-selectin, VCAM-1, and ICAM-1. J Clin Apher.

[41] Pulawski E, Mellwig KP, Brinkmann T (2002). Influence of single low-density lipoprotein apheresis on the adhesion molecules soluble vascular cellular adhesion molecule-1, soluble intercellular adhesion molecule-1, and P-selectin. Ther Apher.

[42] Dlouha D, Blaha M, Blaha V (2017). analysis of circulating miRNAs in patients with familial hypercholesterolaemia treated by LDL/Lp(a) apheresis. Atheroscler Suppl.

[43] Rathouska J, Jezkova K, Nemeckova I (2015). Soluble endoglin, hypercholesterolemia and endothelial dysfunction. Atherosclerosis.

[44] Blazquez-Medela AM, Garcia-Ortiz L, Gomez-Marcos MA (2010). Increased plasma soluble endoglin levels as an indicator of cardiovascular alterations in hypertensive and diabetic patients. BMC Med.

[45] Gallardo-Vara E, Gamella-Pozuelo L, Perez-Roque L (2020). Potential role of circulating endoglin in hypertension via the upregulated expression of BMP4. Cells.

[46] Vitverova B, Blazickova K, Najmanova I (2018). Soluble endoglin and hypercholesterolemia aggravate endothelial and vessel wall dysfunction in mouse aorta. Atherosclerosis.

[47] Dlouha D, Blaha M, Blaha V (2017). analysis of circulating miRNAs in patients with familial hypercholesterolaemia treated by LDL/Lp (a) apheresis. Atherosclerosis Supplements.

[48] Vaverkova H, Tichy L, Karasek D (2019). A case of autosomal recessive hypercholesterolemia caused by a new variant in the LDL receptor adaptor protein 1 gene. J Clin Lipidol.

[49] Blaha M, Cermanova M, Blaha V (2007). Safety and tolerability of long lasting LDL-apheresis in familial hyperlipoproteinemia. Ther Apher Dial.

[50] Solichova D, Melichar B, Blaha V (2001). Biochemical profile and survival in nonagenarians. Clin Biochem.

[51] Blaha M, Kostal M, Lanska M (2013). The decrease of mean platelet volume after extracorporeal LDL-cholesterol elimination. Atheroscler Suppl.

[52] Blaha M, Zadak Z, Blaha V (2009). Extracorporeal LDL cholesterol elimination (25 years of experience in CZ). Atheroscler Suppl.

[53] Blaha M, Pecka M, Urbankova J (2004). Activity of thrombocytes as a marker of sufficient intensity of LDL-apheresis in familial hypercholesterolaemia. Transfus Apher Sci.

